# Thromboembolism Prevention via Transcatheter Left Atrial Appendage Closure with Transeosophageal Echocardiography Guidance

**DOI:** 10.1155/2014/832752

**Published:** 2014-02-11

**Authors:** John Palios, Ioannis Paraskevaidis

**Affiliations:** ^1^Department of Cardiology, Emory University Hospital, 1365 Clifton Road NE, Suite AT 503, Atlanta, GA 30322, USA; ^2^Department of Cardiology, Attikon University Hospital, 1 Rimini Road, Haidari, 12462 Athens, Greece

## Abstract

Atrial fibrillation (AF) is an independent risk factor for stroke. Anticoagulation therapy has a risk of intracerebral hemorrhage. The use of percutaneous left atrial appendage (LAA) closure devices is an alternative to anticoagulation therapy. Echocardiography has a leading role in LAA closure procedure in patient selection, during the procedure and during followup. A comprehensive echocardiography study is necessary preprocedural in order to identify all the lobes of the LAA, evaluate the size of the LAA ostium, look for thrombus or spontaneous echo contrast, and evaluate atrial anatomy, including atrial septal defect and patent foramen ovale. Echocardiography is used to identify potential cardiac sources of embolism, such as atrial septal aneurysm, mitral valve disease, and aortic debris. During the LAA occlusion procedure transeosophageal echocardiography provides guidance for the transeptal puncture and monitoring during the release of the closure device. Procedure-related complications can be evaluated and acceptable device release criteria such as proper position and seating of the occluder in the LAA, compression, and stability can be assessed. Postprocedural echocardiography is used for followup to assess the closure of the LAA ostium. This overview paper describes the emerging role of LAA occlusion procedure with transeosophageal echocardiography guidance as an alternative to anticoagulation therapy in patients with AF.

## 1. Introduction

Atrial fibrillation (AF) is an independent risk factor for stroke. Anticoagulation therapy is effective at reducing the risk of thromboembolic events in AF but necessitates regular blood monitoring, can be disrupted due to frequent interactions with concomitant medications such as antibiotic therapy, and is associated with increased bleeding risk. Therefore, despite demonstrable benefit, alternatives to anticoagulation therapy have long been sought in patients with AF. An emerging option for patients with AF who cannot safely receive anticoagulation has been to obliterate the LAA to remove a potential risk for thrombus formation. Anticoagulation remains the standard of care and has been studied extensively, most recently with the introduction of a new generation of anticoagulant agents for nonvalvular AF. Stroke prevention in patients with AF has largely been based on the use of anticoagulation with warfarin and recently with direct thrombin inhibitor dabigatran and the selective factor X_a_ inhibitors apixaban and rivaroxaban. Anticoagulation nevertheless remains an iatrogenically induced disease with significant associated morbidity and mortality to which patients may be exposed for decades. Furthermore a significant proportion of patients with AF do not receive anticoagulation due to relative or absolute contraindications or patients reluctance. Mechanical alternatives have been based on the assumption that the left atrial appendage (LAA) is the locus for virtually all clots in nonvalvular AF. Several methods have been developed to achieve this by percutaneous or surgical approaches, the primary aim being to exclude blood flow into and out of the LAA [1]. Percutaneous LAA occlusion [2] has the advantage of being a minimally invasive treatment for patients in whom long-term anticoagulation treatment is deemed unsuitable and may be equivalent to treatment with oral anticoagulant agents in those individuals considered at moderate-to-high risk of thromboembolism [3].

## 2. Thromboembolism Prevention with LAA Closure Procedures

Risk of thromboembolic stroke in patients with AF in clinical practice is assessed via the CHADS_2_ score (congestive heart failure, hypertension, age >75 years, diabetes, and stroke) in a scale of 0–6 points: 1 point for each one of congestive heart failure, hypertension, age, and diabetes and 2 points for history of stroke. This score has been updated recently by a 0–9 points score, the CHA_2_DS_2_-Vasc score where 1 point is calculated for each one of congestive heart failure, hypertension, diabetes, vascular disease (myocardial infarction, peripheral artery disease, and aortic plaque), age 65–74 years, and sex category female and 2 points for history of stroke/transient ischemic attack/thromboembolism and age of 75 years and above. Low risk is categorized as a CHA_2_DS_2_-Vasc score of 0, intermediate risk as a score of 1, and high risk as a score ≥2. Increased CHA_2_DS_2_-Vasc score is associated with increased risk of thromboembolic events. Although anticoagulant therapy with warfarin and recently with direct thrombin inhibitor dabigatran and the selective factor X_a_ inhibitors apixaban and rivaroxaban is recommended to reduce stroke risk in AF, alternative strategies are needed for patients who cannot tolerate or who have contraindications to use anticoagulation agents.

**Figure 1:**
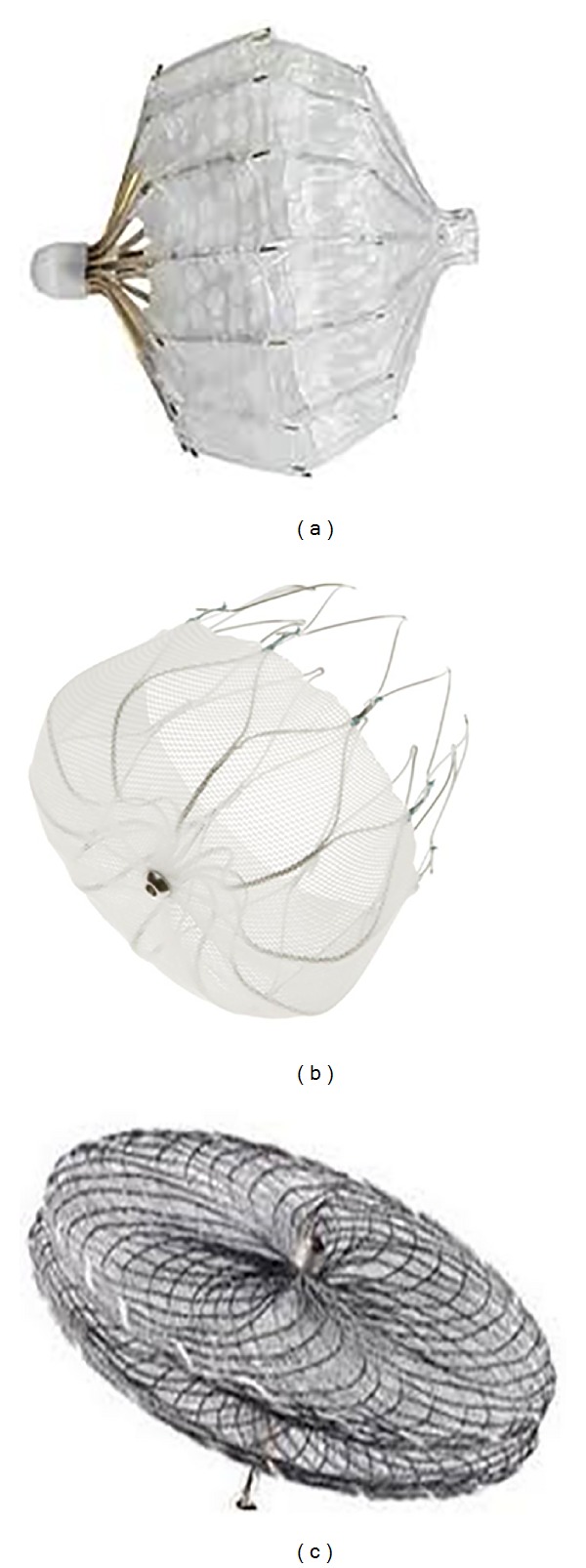
Devices for percutaneous transcatheter LAA closure. (a) The PLAATO (ev3 Endovascular, Inc., North Plymouth, MN, USA) device was the first transcatheter LAA occlusion device implanted percutaneously in patients with atrial fibrillation. (b) The WATCHMAN (Atritech Inc.) LAA occlusion system consists of a parachute-shaped device with a self-expanding nitinol frame structure with a permeable polyester membrane over the atrial side and mid-perimeter fixation barbs to secure it in the LAA. (c) The Amplatzer cardiac plug (AGA Medical Corporation, Golden Valley, MN, USA) device consists of two bodies: a distal anchoring lobe and a proximal sealing disc linked via a flexible central waist.

Occlusion of the LAA has emerged as an alternative treatment option to anticoagulation agents. Currently there are several different percutaneous or minimally invasive approaches to elimination of the LAA as a source of clot embolization (Figure 1). The main characteristics of the currently available devices for percutaneous LAA closure devices are summarized in Table 1. This field originated with the invention of the PLAATO (percutaneous left atrial appendage transcatheter occlusion) device [4], a nitinol and fabric mesh combination that was the precursor to the extensively studied WATCHMAN [5] as well as the AMPLATZER cardiac plug (ACP) [6]. In addition to the ACP, other approaches are described: suture ligation of the LAA (LARIAT device) via a percutaneous subxiphoid transpericardial approach [7] and amputation of the LAA via thoracoscopic use of a widely available stapler/cutter.

The PLAATO implant consists of a spherical nitinol self-expanding metal cage with multiple struts that support an occlusive membrane of expanded polytetrafluoroethylene (an echo-reflective material due to microscopic trapped air), which occludes the lumen of the LAA and promotes healing. It has been out of market for commercial reasons. Devices of different sizes were available, measuring from 15 to 32 mm in diameter. The device selected was usually 20–40% larger than the orifice of the LAA. If the result of device implantation was suboptimal, the device could be collapsed back into the delivery sheath and replaced with another size.

The WATCHMAN System comprises a self-expanding nitinol frame structure with fixation barbs covered with permeable polyester fabric that allows blood to flow into or out of the LAA but excludes passage of thrombi. The shape is semispherical on the left atrium side and tapered towards the appendage side. Device diameters range from the smallest at 21 mm, increasing by 3 mm to a maximum of 33 mm. A size is chosen such that the device measures 10–20% larger than the diameter of the LAA. This system, however, requires anticoagulation for 45 days up to 6-month postimplantation to allow endothelialization and might therefore be unsuitable if anticoagulation is an absolute contraindication. Like the PLAATO, the WATCHMAN occludes the LAA lumen.

The ACP is a transcatheter, self-expanding device constructed from nitinol wire mesh and Dacron patches sewn inside the device and consists of a lobe designed to conform to the inner lumen of the LAA and a disc connected by a central waist. The waist acts as an articulating, compliant connection between the disc and lobe allowing the disc to self-orient to the cardiac wall; the disc is designed to seal the ostium of the LAA. There are 12 stabilizing wires spaced circumferentially around the main disc to improve device placement and retention. It is available in eight lobe sizes from 16 to 30 mm diameter; the size of the chosen device is between 1.5 and 3.4 mm larger than the diameter of the LAA “landing zone.”

Access in all systems is obtained via a transeptal sheath in the femoral vein through which the delivery catheter is deployed. A transeptal puncture is performed and the implant is introduced into the left atrium (LA). The delivery sheath allows injection of contrast in the LAA and proximal to the device to facilitate accurate placement and angiographic assessment of leakage and also allow repositioning and adjustments to be made before final deployment. LAA anatomy tends to be highly variable, often with multiple lobes and therefore no single device is likely to be suitable for all patients.

## 3. The Role of Echocardiography in LAA Occlusion Procedure

### 3.1. Preprocedural Evaluation

**Figure 2:**
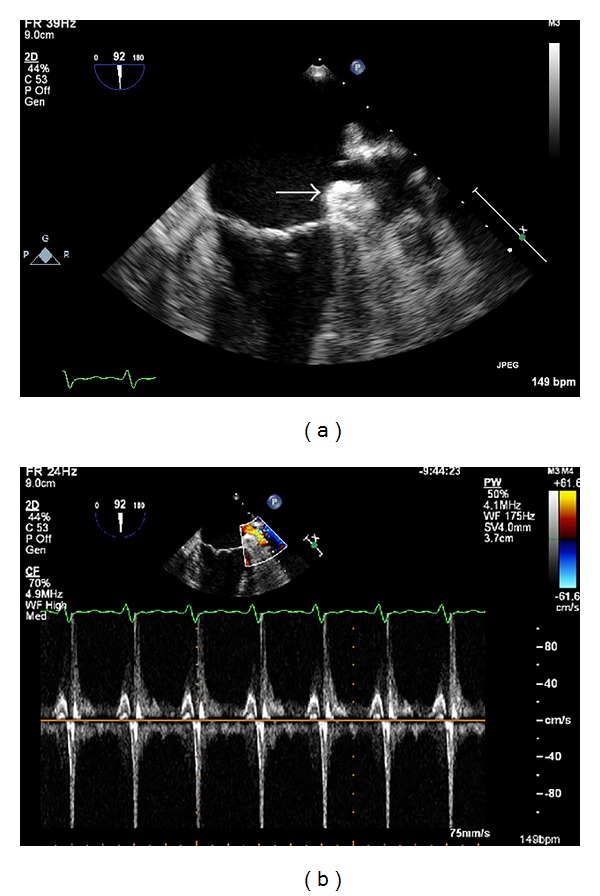
TEE images at mid-esophageal 92 degrees suggestive of a thrombus in LAA in a patient planning for LAA closure. In 2D image (a) a clot is seen in the LAA (white arrow). In pulsed wave Doppler image (b) the LAA wash-out velocity is low (<0.4 m/s). The LAA occlusion procedure was cancelled.

Comprehensive evaluation of LAA cannot be achieved with transthoracic echocardiography. LAA structure and function have traditionally been carried out with transeosophageal echocardiography (TEE) as a result of its close proximity to the esophageal transducer and the high image resolution provided by this technique [8]. TEE assessment is currently the main cardiac imaging modality used to screen suitable candidates for device closure of the LAA [9]. During TEE the LAA is best assessed in the mid-esophageal level starting at 0 degree angle and the rotate in increments of 15–30 degrees angle until the full extend of the appendage is visualized. Two-dimensional (2D) TEE is performed for anatomic evaluation while color flow and pulsed Doppler echocardiography is performed for quantitate measurements of flow patterns. Three-dimensional (3D) TEE is currently successfully performed for further anatomical image acquisition [10]. Although 2D TEE is currently the most commonly used imaging modality for preoperative assessment, the use of 3D TEE provides additive information in cases of complex morphology of the LAA. The main exclusion criterion is the presence of thrombus in the LAA (Figure 2), although the presence of spontaneous echo contrast (SEC) and significant valve disease that could have an impact to the procedure can also be noted. 2D TEE provides comprehensive pre procedural images of the LA and assessment of the ostial dimensions of the LAA in order to determine the size of the occluder device required. Ostial dimensions are made from mid-esophageal views on different probe angles. Typically, a device with a diameter larger than the LAA ostium is chosen to ensure sufficient anchoring for stable positioning. The maximum length of the dominant lobe of the LAA is recorded. Device sizing is crucial to ensure device stability, optimum sealing of the LAA ostium, and minimize the risk of leakage, which could provide a new source of thrombus. Assessment of LA inflow and outflow using Doppler velocity measurements provides baseline evaluation of LA function. The diameter of the left upper pulmonary vein (LUPV) at the point of insertion site into the LA is measured, alongside peak systolic and diastolic pulmonary vein flow using pulse-wave Doppler. Mitral valve anatomy is determined through 2D and 3D TEE imaging. The severity of mitral regurgitation (MR) is assessed visually and by regurgitant jet/LA ratio, vena contracta, proximal isovelocity surface area (PISA) radius, regurgitant fraction, regurgitant orifice area, and regurgitant volume measurements.

### 3.2. Periprocedural Guidance

**Figure 3:**
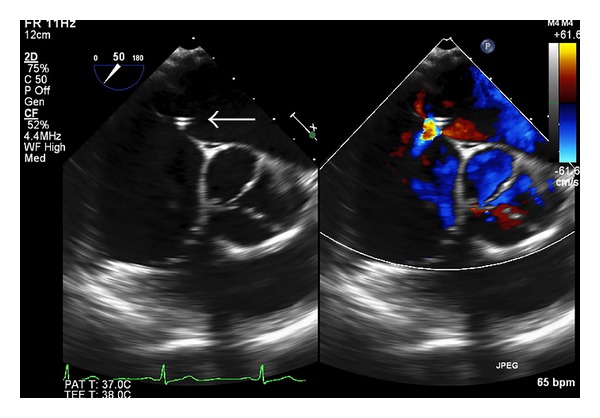
LAA occlusion periprocedural TEE images at mid-esophageal 50 degrees. 2D and color Doppler short axis image of the atrial septal puncture point for the LAA access where the transcatheter LAA occluder will pass from right to left atrium and reach LAA (white arrow). The aortic valve is noted in the center of the image.

**Figure 4:**
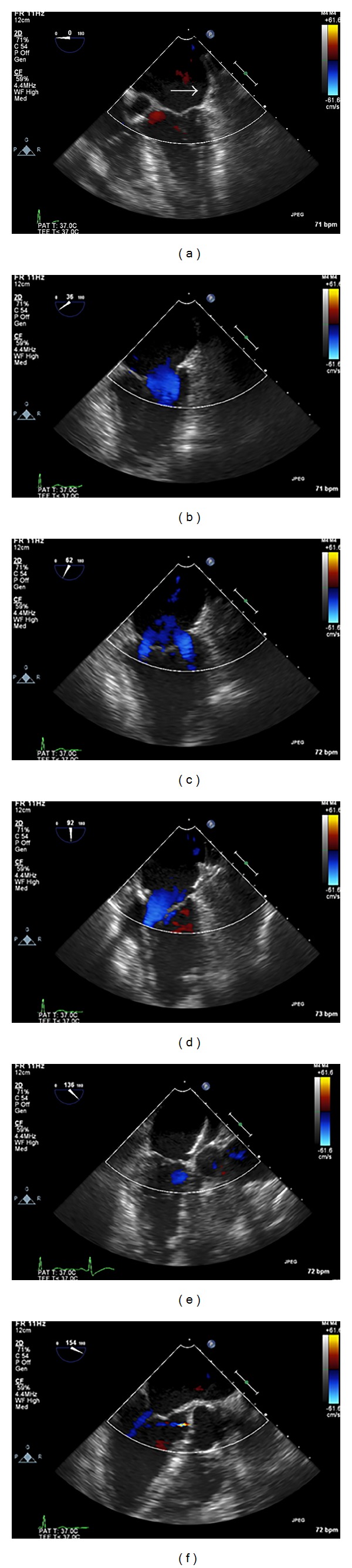
LAA occlusion periprocedural TEE color Doppler images at mid-esophageal 0, 36, 62, 92, and 136 degrees, respectively, ((a)–(f)) for comprehensive evaluation of possible LAA occlusion device leak. The Amplatzer cardiac plug is well seated (white arrow) in the LAA and there is no evidence of perioccluder leak.

Periprocedural echocardiography guidance is essential for the delivery and deployment of the LAA occluder device. The use of fluoroscopy alone to guide device placement appears insufficient, particularly in cases of complex LAA anatomy. The periprocedural echocardiographic modality of choice is multiplane 2D TEE, but intracardiac echocardiography (ICE) using a phased-array system with color Doppler imaging could be an alternative [11]. Both ICE and TEE appear equivalent in their ability to determine LAA dimensions, in confirming the absence of LAA thrombus, and in verifying the location and stability of the occluder device post deployment. The use of real-time 3D TEE may provide additional anatomical information and improved demonstration of the spatial relationship of atrial structures compared with conventional 2D TEE imaging. Once LA thrombus has been excluded, the size and shape of the LAA can be assessed and combined with angiographic measurements; guidance for the device size selection can be provided. With the ACP device, sizing of the landing zone of the anchoring lobe is also measured since it cannot be used if the landing zone is less than 10 mm width. TEE is used in guiding the transeptal puncture (Figure 3), verifying position of the delivery sheath, and aiding delivery and deployment of the device at the LAA ostium. The relationship and orientation of the implant to the LAA and LA wall are also assessed, ensuring that the axis of the device is in alignment with the major axis of the LAA. Color-flow Doppler is used to detect any leaks into the LA (Figure 4), which may suggest an undersized implant and stability of the test observed during traction. A complete seal of the LAA orifice must be confirmed before release. Any interference with surrounding structures resulting in disruption of MV function and pulmonary vein flow (particularly of the LUPV) is noted prior to final irretrievable device deployment. TEE imaging for any procedural complications such as pericardial effusion, thrombus associated with implantation, or device migration or embolization is important.

### 3.3. Postprocedural Surveillance

During follow-up TEE the occluding device is assessed for stability and for evidence of displacement, erosion, or encroachment on surrounding structures, particularly the LUPV and mitral valve. This is suggested by a reduction in diameter of the LUPV at its atrial insertion site compared with baseline, or through detection of turbulent flow. The atrial part of the device is assessed for evidence of any thrombus development. The success of LAA occlusion should be graded according to the presence of any leak around the device margins, detected using color Doppler imaging. Severe leak with multiple jets of free flow is considered Grade 1. Moderate (Grade 2) leak is considered any >3 mm diameter jet noted. Mild leak, with a 1–3 mm diameter jet present, is considered Grade 3. Trace leak, with a <1 mm diameter jet, is considered Grade 4. Grade 5 is considered when no evidence of perioccluder leak is present. Successful LAA occlusion is defined as Grade 3 or higher [8]. Color Doppler imaging over the atrial septum is also used to screen for a persistent shunt following transeptal puncture. Postprocedural LA dimensions are determined and compared with pre procedural measurements.

**Figure 5:**
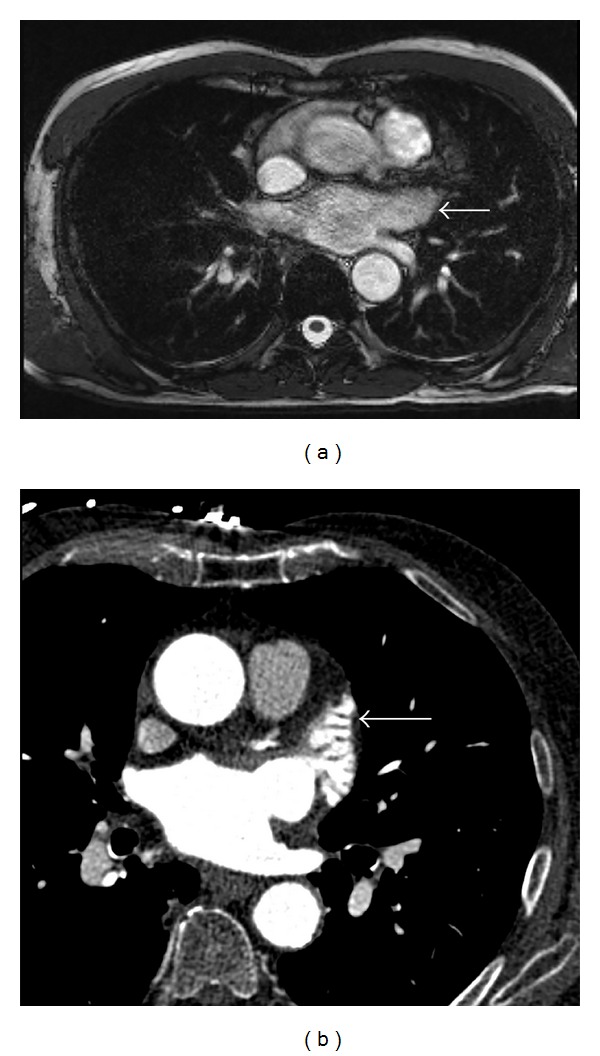
CMR (a) and CCT (b) axial images with contrast of the LAA (white arrows). No thrombus is present in the LAA.

Echocardiography is currently the imaging modality of choice for LAA closure procedures and its role has been extensively reviewed though limited for gaining additional extra cardiac anatomical information. Alternative imaging modalities such as cardiac computed tomography (CCT) and cardiac magnetic resonance imaging (CMR) have been used lately for pre- and post- procedural evaluation of the LA and the LAA (Figure 5). The exact morphology and anatomical variability of the LAA can be assessed with both these techniques and 3D segmented CCT [12] appears to be most effective. Detection of thrombus and therefore evaluate the risk of stroke is feasible through CCT [13] and CMR [14]. CMR has the advantage of nonradiation but is contraindicated in patients with implantable devices such as pacemakers or defibrillators. The use of iodine contrast agent should be taken into account for patients with known allergies and end stage renal disease. CCT can be used in patients with implantable devices; it has higher spatial resolution and relatively lower cost, but he need for contrast agent and exposure to radiation should be taken into account as well.

## 4. Conclusions

AF is a known risk factor for thromboembolism. Thromboembolism prevention strategies traditionally refer to anticoagulation therapy which has a risk of intracerebral hemorrhage. The majority of thrombus in patients with nonvalvular AF is seen in LAA. Different LAA closure procedures have been recently developed and appear to be an alternative to anticoagulation therapy. Transcatheter LAA closure with the use of occluder devices is an emerging method. The value of TEE imaging during transcatheter procedures has been demonstrated in a variety of settings [15], and in the case of percutaneous LAA occlusion TEE guidance is of paramount importance: it is used to measure the diameter of the LAA ostium (determining device size), to demonstrate proper positioning of the delivery sheath in the LAA following transeptal puncture, and to confirm proper orientation of the device within the LAA and occlusion of its ostium before being anchored. Procedure-related complications can also be evaluated. Post-procedural TEE is used for follow-up to assess the successful closure of the LAA ostium. Percutaneous occlusion of the LAA under transeosophageal echocardiography guidance is currently an alternative treatment option to anticoagulation agents for patients with AF.

## Figures and Tables

**Table 1:** Summary of main characteristics of the currently used transcatheter LAA occlusion devices.

Device name	Size	Construction	Transcatheter delivered	Self-expanding	Blood flow	Temporary anticoagulation
WATCHMAN	21–33 mm	Nitinol/polyester	Yes	Yes	Yes	Yes
ACP	16–30 mm	Nitinol/Dacron	Yes	Yes	No	No
